# Primary tuberculous otomastoiditis complicated with Bezold’s, postauricular, and subdural abscesses: a case report and review of the literature

**DOI:** 10.1093/jscr/rjad176

**Published:** 2023-04-22

**Authors:** Nawaf Aljehani, Abdulazeez Alzailaie, Jihad Nassar

**Affiliations:** College of Medicine, King Saud bin Abdulaziz University for Health Sciences, Riyadh, Saudi Arabia; College of Medicine, King Saud bin Abdulaziz University for Health Sciences, Riyadh, Saudi Arabia; Division of Otolaryngology-Head and Neck Surgery, Department of Surgery, King Abdulaziz Medical City, Ministry of National Guard Health Affairs, Riyadh, Saudi Arabia

**Keywords:** Mastoiditis, TB otomastoiditis, Bezold’s abscess, Subdural abscess, Postauricular abscess

## Abstract

Primary tuberculous otomastoiditis is a rare condition. Mastoiditis is an infection of the mastoid part of the temporal bone that often happens as a complication of otitis media. The spread of infection from the middle ear and mastoid to adjacent structures might result in rare but serious complications. We present a case of an 8-year-old female with recurrent episodes of acute otitis media and foul-smelling yellowish discharge with hearing loss. Imaging revealed multiple abscesses. Intraoperatively, samples were taken from the abscesses and sent for complete analysis revealing a tuberculous infection. A diagnosis of primary *Mycobacterium tuberculosis* (MTB) otomastoiditis was made using MTB polymerase chain reaction from the Bezold’s abscess. The patient was started on anti-MTB therapy. Follow-up imaging showed resolution of abscesses and otomastoiditis. An indolent course of otitis media along with poor response to conventional antibiotics regimen should raise the suspicion of rare and unusual infectious etiologies.

## INTRODUCTION

Tuberculous otitis media is a rare extrapulmonary manifestation of *Mycobacterium tuberculosis* (MTB). Primary tuberculous otomastoiditis is a rare condition as it comprises only 0.04–0.9% of all chronic otitis media cases [1–4]. Mastoiditis is an infection of the mastoid part of the temporal bone that happens as a complication of undertreated otitis media. The spread of middle ear infection to adjacent structures might result in serious complications, among them CNS infections or deep neck abscesses as observed in our case. Bezold’s abscess (BA) occurs when the infection erodes through the mastoid cortex medial to the attachment of sternocleidomastoid (SCM) and posterior belly of the digastric muscles [5, 6]. The co-occurrence of MTB otomastoiditis and BA is a rare entity described in only one case report [7]. In this paper, we present a case of primary tuberculous otomastoiditis complicated with Bezold’s, postauricular and subdural abscesses in an otherwise healthy pediatric patient. We aim to shed light on the indolent course of the disease to be able to reach an early diagnosis and prevent life-threatening complications.

## CASE REPORT

**Figure 1:**
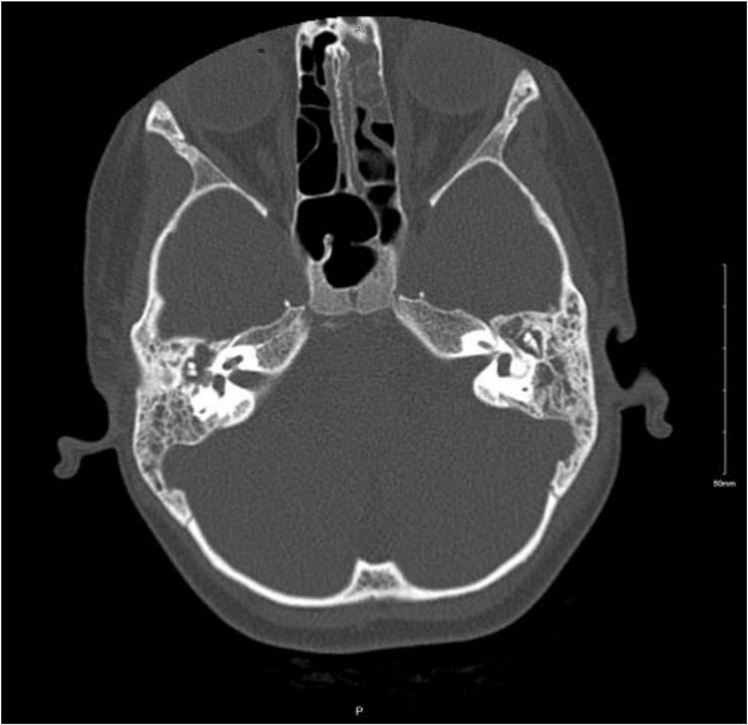
Non-enhanced axial CT scan of temporal bone showing bony remodeling suggestive of chronic otomastoiditis.

An 8-year-old female patient presented with recurrent episodes of acute otitis media (AOM) and foul-smelling yellowish discharge from the left ear with hearing loss, after receiving multiple courses of antibiotics with only slight improvement. Otoscopic examination showed left small central perforation with minimal granulation tissue and right middle ear effusion. The patient was afebrile with no constitutional symptoms. A CT scan of the temporal bone revealed signs of bilateral mastoid and middle ear opacification with left mastoid cortical erosion, an overlying soft tissue swelling, and a parapharyngeal hypodense collection that likely represents an abscess (Fig. 1). Therefore, the patient was hospitalized for further investigations.

**Figure 2:**
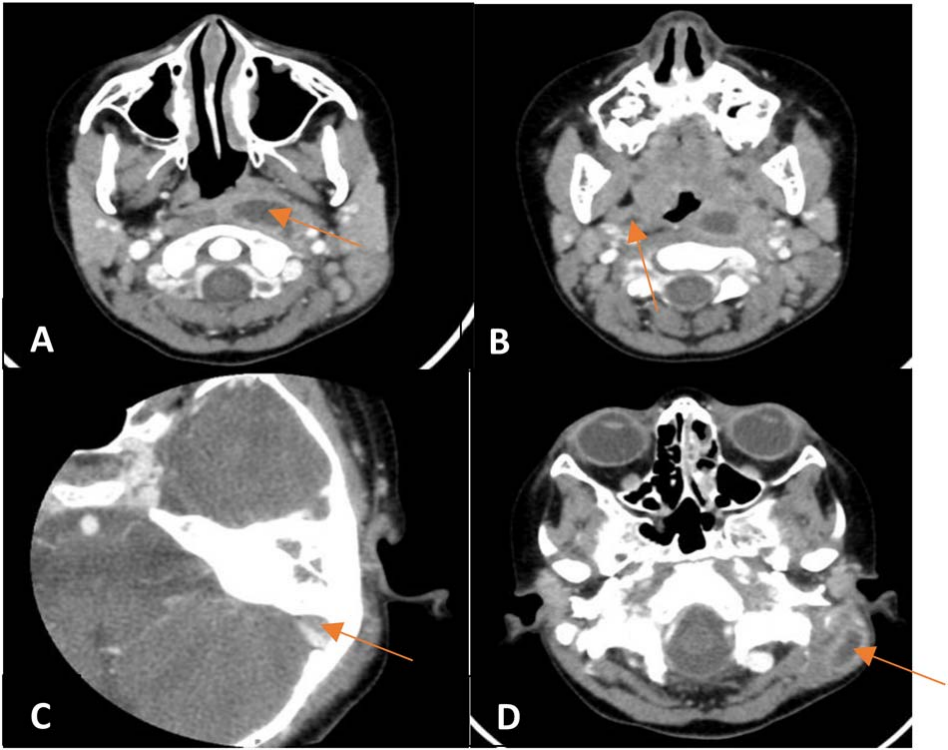
Enhanced CT scan of the temporal bones and neck showing (**A**) a left parapharyngeal space fluid collection with enhancing walls, (**B**) a small right parapharyngeal collection with minimally enhancing walls, (**C**) an intracranial extension of mildly enhancing lesion to subdural space and (**D**) a left mastoid bone showing lateral wall cortical disruption with adjacent retro-auricular irregular collection with enhancing walls.

A contrasted CT scan (Fig. 2) confirmed the presence of a left parapharyngeal abscess (2.7 × 1.2 × 3.7 cm) with a smaller right abscess (0.8 × 0.5 × 1.3 cm). Also, a left subdural abscess (1.1 × 0.4 cm) and a postauricular irregular collection at the proximal SCM measuring 1.9 × 0.9 × 2.8 cm were noted.

**Figure 3:**
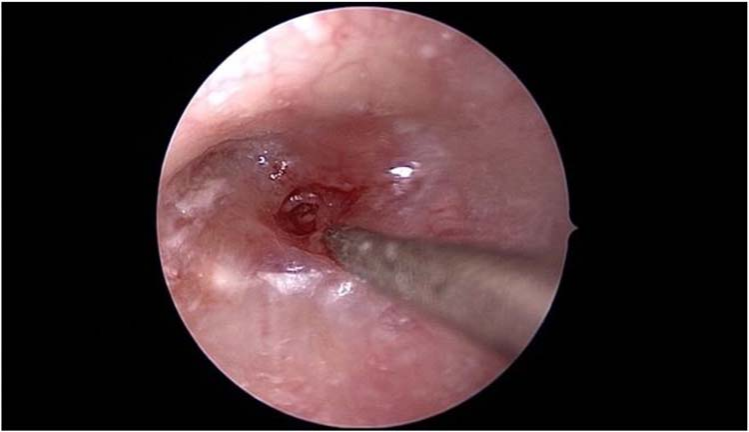
Intraoperative endoscopic view of the left middle ear cavity filled with granulation tissue.

In view of the findings, the patient was taken to the operating room for left tympanomastoidectomy with bilateral tympanostomy tubes insertion and intraoral drainage of the left BA. Intraoperatively, the left tympanic membrane was already healed; however, it was significantly thickened and inflamed during myringotomy. The middle ear space was full of granulation tissue with no purulent discharge that could be retrieved (Fig. 3).

The mastoid bone had no bony erosion of the septations. There was only granulation tissue in the antrum, sigmoid sinus area and the tip air cells. Also, necrotic tissue at the tip of the mastoid was noticed. The sigmoid sinus was partially dehiscent covered with granulation tissue. Similarly, inflammation and granulation were seen while performing the right myringotomy. The left parapharyngeal abscess was drained using an 18-gauge needle aspiration followed by a small incision and thorough irrigation of the abscess.

Intraoperative samples taken from the abscesses were sent for complete analysis, including MTB polymerase chain reaction (PCR). Results from the MTB PCR came back positive. Then, a full MTB work-up was initiated to exclude pulmonary MTB. The patient was switched to anti-MTB therapy (Rifampin 300 mg, Isoniazid 200 mg, Pyrazinamide 700 mg and Ciprofloxacin 300 mg). With the absence of pulmonary tuberculosis, the patient was discharged with a diagnosis of primary MTB otomastoiditis complicated with Bezold’s, postauricular, and subdural abscesses. Meanwhile, more investigations were done ruling out the presence of HIV or primary immune deficiency.

Following the completion of anti-MTB regimen, the patient developed a persistent right tympanic membrane perforation and left middle ear effusion after the tympanostomy tubes extrusion, which will be addressed at a later stage. Pure tone audiometry revealed right moderate conductive hearing loss with left mild conductive hearing loss. The patient was fitted with hearing aids. Follow-up imaging showed resolution of abscesses and otomastoiditis with a residual mastoid air cells partial opacification.

## DISCUSSION

A common presentation of AOM is otorrhea which is usually accompanied by fever unlike otomastoiditis which presents with significant pain, postauricular swelling and tenderness [7]. Also, it shares the same causative organisms of AOM with *Streptococcus pneumoniae*, *Haemophilus influenza* and *Moraxella catarrhalis* being the most common, respectively [7]. On this basis, our patient was treated with the appropriate antibiotics to cover such organisms. However, patient’s lack of response raised the suspicion for cholesteatoma as a differential diagnosis. Such a differential was similarly documented in few case reports [8–10]; however, it was excluded in our case. The diagnosis of primary MTB infection explained the poor response to common antibiotics and the subclinical presentation. The presence of such CT findings along with an indolent course of the disease should shift the suspicion toward rare causes, specifically MTB [9]. Primary tuberculous infection of the middle ear is an extremely rare entity in immunocompetent individuals [7]. Therefore, our patient was worked up for an underlying immune deficiency and later ruled out.

The MTB work-up initiated in our case was negative. Nevertheless, it has been reported previously that real-time PCR can detect MTB in 28.6% of the cases with a negative AFB smear [11]. Similarly, the diagnosis in our case was made based on the MTB PCR of the abscess aspirate. Primary extrapulmonary tuberculous infections, specifically of the middle ear, are rarely seen in clinical practice. In the literature, a few articles reported such a condition [12–14]. Tuberculous infections should be suspected whenever the optimal regimen fails to meet the expected outcomes. A combination of radiological and laboratory assessment is critical for a comprehensive diagnosis and management.

## CONCLUSION

Primary tuberculous otomastoiditis is rarely seen in modern medicine. A chronic and indolent presentation of otitis media with poor response to conventional antibiotics regimen should raise the suspicion of rare and unusual infectious etiologies such as MTB. A high index of clinical suspicion is required to narrow down the differential diagnosis to establish an early diagnosis and initiate the appropriate treatment to prevent serious complications.

## Data Availability

The data underlying this article will be shared on reasonable request to the corresponding author.

## References

[1] Vaamonde P , Castro C, García-Soto N, Labella T, Lozano A. Tuberculous otitis media: a significant diagnostic challenge. Otolaryngol Head Neck Surg 2004;130:759–66. 15195064 10.1016/j.otohns.2003.12.021

[2] Sens PM , Almeida CIR, Valle LO do, Costa LHC, Angeli MLS. Tuberculose de orelha, doença profissional? Rev Bras Otorrinolaringol 2008;74:621–7.

[3] Ma KH , Tang PS, Chan KW. Aural tuberculosis. Am J Otol 1990;11:174–7. 2343901

[4] Krause E , Leunig A, Klopstock T, Gürkov R. Treatment of essential palatal myoclonus in a 10-year-old girl with botulinum neurotoxin: temporal bone histopathology and clinical extrapolation. Otol Neurotol 2006;27:672–5. 16868515 10.1097/01.mao.0000224085.08344.50

[5] Castillo M , Albernaz VS, Mukherji SK, Smith MM, Weissman JL. Imaging of Bezold’s abscess. AJR Am J Roentgenol 1998;171:1491–5. 9843276 10.2214/ajr.171.6.9843276

[6] Bezold F. Ein neuer Weg für Ausbreitung eitriger Entzündung aus den Räumen des Mittelohrs auf die Nachbarschaft und die in diesem Falle einzuschlagende Therapie. Dtsch Med Wochenschr 1881;7:381–5.

[7] Singh A , Irugu DVK, Verma H, Thakar A. Atypical presentation of aural tuberculosis with complication. BMJ Case Rep 2018;2018:bcr-2017-222482. 10.1136/bcr-2017-222482 PMC5847930 29523606

[8] Aziz A , Md Daud MK. Primary middle ear tuberculosis mimicking cholesteatoma. Malays Fam Physician 2020;15:44–6. 32284804 PMC7136677

[9] Cavallin L , Muren C. CT findings in tuberculous otomastoiditis. A case report. Acta Radiol 2000;41:49–51. 10665870

[10] Munoz A , Ruiz-Contreras J, Jimenez A, Maté I, Calvo M, Villafruela M, et al. Bilateral tuberculous otomastoiditis in an immmunocompetent 5-year-old child: CT and MRI findings (2009: 3b). Eur Radiol 2009;19:1560–3. 19440720 10.1007/s00330-008-1130-7

[11] Garg P , Garg MK, Agarwal N. Comparison of histopathology, acid fast bacillus smear and real-time polymerase chain reaction for detection of *Mycobacterium tuberculosis* in anal fistula in 161 patients: a prospective controlled trial. Int J Mycobacteriol 2016;5:S208–9. 28043558 10.1016/j.ijmyco.2016.09.055

[12] Araujo MF , Pinheiro TG, Raymundo IT. Tuberculous otitis media. J Int Adv Otol 2011;7:413–7.

[13] Bruschini L , Ciabotti A, Berrettini S. Chronic tuberculous otomastoiditis: a case report. J Int Adv Otol 2016;12:219–21. 27716611 10.5152/iao.2016.2097

[14] Jayakody N , Faoury M, Hellier W, Ismail-Koch H, Patel S, Burgess A. A rare presentation of a paediatric patient with acute otomastoiditis media caused by *Mycobacterium tuberculosis* resulting in intracranial complications. J Surg Case Rep 2019;2019:rjz093. 30967931 10.1093/jscr/rjz093 PMC6446536

